# Reasons for Crown Failures in Primary Teeth: Systematic Review and Meta-Analysis

**DOI:** 10.2196/57958

**Published:** 2025-05-01

**Authors:** Stephan Lampl, Deepa Gurunathan, Deepak Mehta, Krithikadatta Jogikalmat

**Affiliations:** 1 Department of Pediatric Dentistry, Saveetha Dental College and Hospitals, Saveetha Institute of Medical and Technical Sciences, 162, Poonamallee High Rd, Velappanchavadi, Chennai, 600077, India; 2 Department of Cariology, Saveetha Dental College and Hospitals, Saveetha Institute of Medical and Technical Sciences, Chennai, India; 3 Department of Operative Dentistry, Tohoku Graduate School of Dentistry, Tohoku University, Sendai, Japan

**Keywords:** primary teeth, pediatrics, children, biological complications, technical complications, survival rates, dental, oral, dentist, synthesis, review methods, search, systematic, meta-analysis, complication, crown

## Abstract

**Background:**

Understanding long-term retention rates and complications associated with different materials for fabricating pediatric crowns for primary teeth is crucial for material selection and optimizing clinical outcomes.

**Objectives:**

This systematic review aimed to descriptively analyze the crown-retention rates and complications associated with crown retention, as well as the biological and technical complications of pediatric crowns, for primary teeth. The meta-analysis reported herein was performed to estimate long-term (3-year and 5-year) retention rates of these pediatric crowns fabricated using various materials.

**Methods:**

Using the PICOS (Population, Intervention, Comparison, Outcomes, and Study design) paradigm, a systematic search was conducted between July and August 2023 in the Cochrane, Embase, and PubMed databases to identify randomized controlled trials (RCTs) and clinical (prospective and retrospective) studies reporting retention rates, complications of crown retention, and biological and technical complications. After selecting studies with a predefined set of selection criteria, data from included studies were used for a systematic review aimed at a descriptive analysis of factors associated with the failure of crowns for primary teeth. Data from the included RCTs were used for meta-analysis, wherein 3-year and 5-year crown-retention rates were estimated using Poisson regression models.

**Results:**

This systematic review included 13 RCTs and 5 clinical studies on dental crowns for primary teeth, involving 454 children (1172 crowns) in RCTs and 810 children (2667 crowns) in clinical studies. The median follow-up durations were 12 months for RCTs and 20.8 months for clinical studies, with a 10.6% (124/1172) dropout rate in RCTs. Meta-analysis of pooled 5-year retention rates for different crown materials revealed the following retention rates: 88.90% for compomer crowns, 92.18% for composite resin crowns, 90.30% for resin-modified glass ionomer cement (RMGIC) crowns, and 97.88% for stainless steel crowns. Additionally, strip crowns exhibited a retention rate of 83.48%, while zirconia crowns had a retention rate of 97.09%. Poisson regression estimated 3-year and 5-year crown-retention rates, indicating good outcomes across materials. Complications included secondary caries (up to 21.8% in zirconia crowns) and marginal adaptation issues (up to 22.2% in compomer crowns). These findings highlight material-specific considerations necessary for optimizing outcomes in pediatric dental crown treatments.

**Conclusion:**

While retentive complications such as chipping, material loss, and fractures do occur across materials, compomer, composite resin, stainless steel, strip, and zirconia crowns all have clinically acceptable retention rates. However, the differences in biological and technical complications between materials may provide insights for selecting appropriate materials for pediatric crowns based on clinical considerations.

## Introduction

Dental caries is a highly prevalent chronic disease affecting millions of children worldwide, leading to pain, infection, and difficulties in eating and speaking [1-3]. Untreated caries of the primary teeth may affect over 621 million children globally [1]. Early intervention with fillings is essential to manage caries, but in cases where the tooth structure is significantly compromised, pediatric dental crowns become necessary to preserve the tooth’s functionality and prevent premature loss [4].

Several studies have documented the usefulness and efficacy of materials such as stainless steel, zirconia, composite resin, and polycarbonate materials for fabricating pediatric dental crowns [5-8]. Despite notable advancements in dental materials and restorative techniques, various biological and technical factors persist in influencing the longevity and acceptability of pediatric dental crowns [8]. Biological factors such as the oral microbiome, the presence of cariogenic bacteria, and the immune response play significant roles in the success or failure of pediatric dental crowns [8]. Additionally, technical factors including crown preparation, cementation methods, and occlusal adjustment can impact the longevity and performance of these restorations [8]. Understanding these factors is crucial for clinicians to make informed decisions regarding treatment planning and material selection.

Chisini et al [8], in their systematic review, highlighted that composite resin crowns exhibited the lowest annual failure rate (1.7% to 12.9%), while stainless steel crowns had the highest success rate (96.1%). This report [8], published in 2018, identified secondary caries as the main reason for the failure of pediatric crowns and recommended an anticariogenic, health-promoting approach. Furthermore, several systematic reviews (published between 2021 and 2023) have also reported on the efficacy of individual restorative materials such as stainless steel, zirconia, and prefabricated crowns [9-11]. Since the publication of these systematic reviews, several randomized controlled trials (RCTs) have been published, warranting an updated systematic review to include these additional articles.

In the context of overall public health, ensuring the longevity and success of pediatric dental crowns not only addresses the immediate dental needs of children but also contributes to their overall health and well-being [12,13]. Improving the success rates of these restorations can reduce the burden of dental diseases and associated health care costs, enhancing the quality of life for affected children. On the premise of this background, this systematic review and meta-analysis aims to provide a comprehensive qualitative assessment of crown-retention rates and the impact of technical and biological factors on crown success and acceptability for primary teeth.

## Methods

### Overview

The protocol for this systematic review and meta-analysis [14] was registered with PROSPERO (CRD42023442266) and conducted in accordance with the MOOSE (Meta-Analysis of Observational Studies in Epidemiology) and PRISMA-P (Preferred Reporting Items for Systematic Review and Meta-Analysis Protocols) guidelines [15,16].

### Eligibility Criteria

RCTs and clinical studies (prospective and retrospective) evaluating crowns fabricated using stainless steel, zirconia, composite resin, compomer, and resin-modified glass ionomer cement (RMGIC) were included. The predefined inclusion criteria for this systematic review encompassed studies with an English abstract that evaluated crown restorations in pediatric patients aged 1-10 years, reporting crown-retention data, reasons for retentive loss, and technical and biological complications. Qualitative interviews, quasi-experimental studies, single-case studies, and series of single-case studies were excluded. Additionally, articles based on conference abstracts and dissertations were excluded.

Biological factors in this analysis included secondary caries and periodontal pathology (including the periodontal index, which assesses the severity of periodontal disease). Clinical parameters included the gingival index (evaluating gingival inflammation) and other periodontal health indicators. Technical factors included anatomic form, marginal adaptation, color match, surface texture, and wear on the opposing tooth. Success rates, if reported, were also included for the purposes of this systematic review and meta-analysis.

### Data Sources and Search Strategy

Using a database-appropriate search strategy, electronic databases, including Cochrane, Embase, and PubMed (MEDLINE), were systematically searched to identify RCTs and clinical studies (prospective and retrospective) based on the eligibility criteria described above. The PICOS (Population, Intervention, Comparison, Outcomes, and Study design) format for this systematic review and meta-analysis was as follows:

- Population: children with primary tooth decay
- Intervention: crown restorations
- Comparators: materials such as stainless steel, zirconia, composite resin, compomer, and RMGIC
- Outcomes: crown retention, as well as technical and biological factors
- Study design: RCTs and clinical studies (prospective and retrospective)

Multimedia Appendix 1 presents the search syntax used for searching the electronic databases. The search strategy was considered adequate to reduce the risk of selection and detection bias.

### Selection of Studies

The search results were imported into Zotero, and duplicates were removed to create a virtual library. Study selection was conducted in 2 stages. In the first stage, 2 independent reviewers (SL and KJ) screened the titles and abstracts of identified studies to determine their eligibility. In the second stage, the same 2 reviewers independently assessed the full-text articles of potentially relevant studies to confirm whether they met the eligibility criteria. Quality assessment of included RCTs and prospective and retrospective clinical studies was also independently performed by SL and KJ. Any disagreements at any stage, including study selection, quality assessment, and data extraction, were resolved through discussion. If consensus could not be reached, a third expert (DG) was consulted, with DG making the final decision.

### Data Extraction

Data extraction was independently conducted by 2 reviewers (SL and KJ) using a standardized data extraction form. Extracted data included author details, year of publication, number of children, age, number of teeth, tooth type, crown material, dropout, follow-up duration, crown retention, and retention complications including biological and technical complications. Discrepancies in data extraction were resolved through discussion between the 2 reviewers. If consensus could not be reached, a third expert (DG) was consulted to make the final decision.

### Risk-of-Bias Assessments

The Risk of Bias 2 tool developed by the Cochrane Collaboration was used to assess the quality of included RCTs [17]. Two independent reviewers evaluated various bias domains in RCTs, including randomization, allocation concealment, blinding of participants and personnel, blinding of outcome assessment, incomplete outcome data, selective reporting, and other sources of bias. After assessing each included RCT for risk-of-bias domains specified by the Risk of Bias 2 tool, an overall risk-of-bias judgment (low, some concerns, or high) was assigned to each trial [17]. A checklist proposed by Moga et al [18] was adapted for assessing the risk of bias in the included prospective and retrospective clinical studies. Only studies with a moderate or low risk of bias were included in this analysis. Furthermore, a funnel plot was generated to assess for any publication bias.

### Meta-Analytic Approach

For quantitative analysis (meta-analysis), retention was defined as the number of crowns that were in situ, regardless of technical and biological complications. Total exposure time refers to the cumulative duration that all crowns remain in the mouth across all included studies. It is calculated by summing the duration each crown is in place for each study and then aggregating these times across all studies. Failure rates resulting from retentive complications were calculated by dividing the number of failures by the total exposure time. Exposure time for each included study was calculated by summing the exposure time for all restorations. A Poisson regression model was used to analyze the calculated rates. Proportions of crowns retained at 3 years and 5 years were estimated with an assumption of constant event rates. The Pearson goodness-of-fit test was performed to assess heterogeneity across studies and to determine whether a fixed-effects or random-effects model should be used. Since no statistically significant heterogeneity was detected in any of the individual groups and rates (*P*>.05), a fixed-effects Poisson regression model was used to estimate the parameters. A *P* value <.05 was considered significant. All analyses were performed using R statistical software (version 4.1.2; R Core Team).

## Results

### Selection of Studies and Risk-of-Bias Assessments

**Figure 1.**
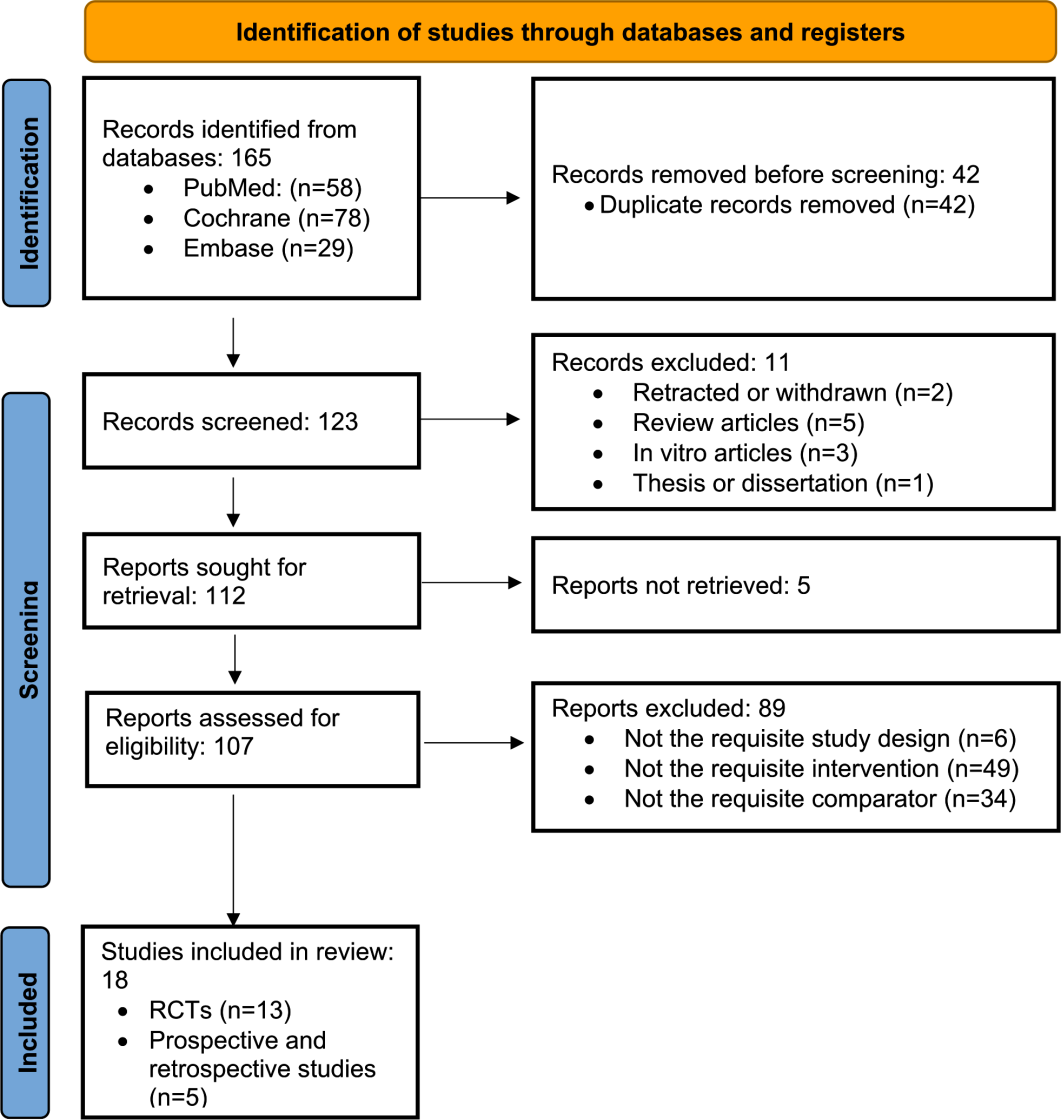
PRISMA (Preferred Reporting Items for Systematic Reviews and Meta-Analyses) flow diagram for selection of studies included in the systematic review and meta-analysis. RCT: randomized controlled trial.

A total of 13 RCTs and 5 clinical studies (prospective and retrospective) were included in this systematic review [19-31]. Figure 1 presents the PRISMA (Preferred Reporting Items for Systematic Reviews and Meta-Analyses) flow diagram illustrating the study selection process. Tables1  2 provide the risk-of-bias assessments for the included RCTs and clinical studies, respectively.

**Table 1.** Risk-of-bias assessments of the included randomized controlled trials using the Cochrane Risk of Bias tool.

Authors	Selection bias		Performance bias	Detection bias	Attrition bias	Reporting bias	Other bias
	Random sequence allotment	Allocation concealment	Blinding of participants and personnel	Blinding of outcome assessment	Incomplete outcome data	Selective reporting	Funding or conflicts of interest
Zulekha et al [19]	+++	+^a^	++^b^	+++^c^	+++	+++	+++
Vaghela et al [20]	+++	+	+	+	+++	+++	+++
Talekar et al [21]	+++	++	++	+++	+++	+++	+++
Hanafi et al [22]	+++	+++	+++	+++	+++	+++	+++
Güçlü et al [23]	+++	+	+	+	+++	+++	+++
Nischal et al [24]	+++	+	+	+	+++	+++	+++
Mathew et al [25]	+++	+	+	+	+++	+++	+++
Gill et al [26]	+++	++	++	+++	+++	+++	+++
Taran and Kaya [27]	+++	+	+	+	+++	+++	+++
Donly et al [28]	+++	++	++	+++	+++	+++	+++
Bektas Donmez et al [29]	+++	++	++	+++	+++	+++	+++
Sengul and Gurbuz [30]	+++	+	+	+	+++	+++	+++
Walia et al [31]	+++	+	+	+	+++	+++	+++

a +: high risk of bias.

b ++: moderate risk of bias.

c +++: low risk of bias.

**Table 2.** Risk-of-bias assessments of the included prospective and retrospective clinical studies.

Authors	Domain 1: study design	Domain 2: population	Domain 3: intervention	Domain 4: outcomes	Domain 5: statistical analysis	Domain 6: results or conclusion	Domain 7: competing interests	Overall
Prabhu et al [32]	++^a^	++	++	++	++	++	++	++
Alhissan and Pani [33]	++	++	++	++	++	++	++	++
Holsinger et al [34]	++	++	++	++	++	++	–^b^	+^c^
Bücher et al [35]	++	++	++	++	++	++	++	++
Daou et al [36]	++	++	++	++	++	++	++	++

a ++: low risk of bias.

b –: serious risk of bias.

c +: moderate risk of bias.

### Qualitative Analysis of the Included Studies

Table 3 summarizes the 13 included RCTs, presenting data on crown retention and retentive complications [19-31]. These RCTs were conducted between 2014 and 2022 and recruited 454 children who received a total of 1172 crowns [19-31]. The follow-up duration ranged from 6 to 36 (median 12, IQR 9) months, with a cumulative dropout rate of 10.6% (124/1172 crowns). Table 4 outlines the 5 included clinical studies, reporting data on crown retention and retentive complications [32-36]. These clinical studies were conducted between 2008 and 2020 and included data from 810 children, with a follow-up duration ranging from 12 to 24 (median 20.8, IQR 5) months [32-36].

**Table 3.** Summary of included randomized controlled trials along with their crown-retention data.

Authors	Children, n	Age (years)	Tooth type	Number of teeth; crown material	Dropout, n (%)^a^	Follow-up duration (months)	Crown retention, n/N (%)	Retention complications
Zulekha et al [19]	25	3 to 5	Incisors	25; one shade composite resin 25; composite resin	0 (0) 0 (0)	12 12	25/25 (100) 25/25 (100)	None None
Vaghela et al [20]	31	3 to 6	Incisors	55; strip crowns 47; zirconia	4 (7) 2 (4)	9 9	44/55 (80.4) 46/47 (97.8)	Chipping (4); material loss (6) Complete loss of crown (1)
Talekar et al [21]	30	4 to 9	Molars	33; glass-reinforced resin 33; zirconia	1 (3) 1 (3)	18 18	29/33 (87.8) 31/33 (93.9)	Chipped (13); complete loss of crown (1) None
Hanafi et al [22]	44	5 to 9	Molars and incisors	31; CCZC^b^ 32; NZC^c^	1 (3) 1 (3)	6 6	31/31 (100) 31/32 (96.9)	None Crown fracture (1)
Güçlü et al [23]	26	1 to 10	Molars and incisors	11; strip crowns 22; zirconia 13; stainless steel	4 (15) 4 (15) 4 (15)	6 6 6	11/11 (100) 19/22 (86.4) 13/13 (100)	None Decementation (3) None
Nischal et al [24]	45	Not known	Incisors	15; strip crowns 15; zirconia 15; luxa	Not known Not known Not known	9 9 9	12/15 (80) 15/15 (100) 12/15(80)	Loss of bulk (2) None Loss of bulk (2)
Mathew et al [25]	30	6 to 8	Molars	30; zirconia 30; stainless steel	Not known Not known	36 36	30/30 (100) 30/30 (100)	None None
Gill et al [26]	49	2 to 4	Incisors	70; strip crowns 70; zirconia 80; stainless steel	22 (31) 30 (43) 33 (41)	12 12 12	55/70 (79) 67/70 (95) 80/80 (100)	Partial material loss (6); complete material loss (3) Partial material loss (1) None
Taran and Kaya [27]	13	6 to 9	Molars	26; zirconia 26; stainless steel	Not known Not known	12 12	25/26 (98) 24/26 (92.3)	Decementation (2) None
Donly et al [28]	50	3 to 7	Molars	50; zirconia 50; stainless steel	Not known Not known	24 24	50/50 (100) 50/50 (100)	None None
Bektas Donmez et al [29]	31	4 to 7	Molars	31; RMGIC^d^ 31; compomer 31; composite resin	2 (6) 1 (3) 4 (13)	18 18 18	28/31 (90.3) 31/31 (100) 25/31 (80.6)	Poor anatomic form (3) None Poor anatomic form (3)
Sengul and Gurbuz [30]	41	5 to 7	Molars	40; hybrid composite resin 32; RMGIC 36; compomer 38; giomer	0 (0) 0 (0) 0 (0) 0 (0)	24 24 24 24	37/40 (92.5) 29/32 (90.3) 28/36 (77.8) 34/38 (89.5)	Crown fracture (3) Crown fracture (3) Crown fracture (8) Crown fracture (4)
Walia et al [31]	39	3 to 5	Incisors	43; strip crowns 43; zirconia 43; stainless steel	7 (16) 5 (12) 6 (14)	6 6 6	34/43 (78) 43/43 (100) 41/43 (95)	Partial material loss (2); complete loss of crown (7) None Partial material loss (2)

a Percentages use the number of teeth as the denominator.

b CCZC: conventional chairside zirconia crown.

c NZC: novel zirconia crown.

d RMGIC: resin-modified glass ionomer cement.

**Table 4.** Summary of included prospective and retrospective studies along with their crown-retention data.

Authors	Children, n	Age (years)	Tooth type	Number of teeth; crown material	Follow-up duration (months)	Crown retention, n/N (%)	Complications
Prabhu et al [32]	60	6 to 10	Molars	30; stainless steel 30; zirconia	24 24	30/30 (100) 30/30 (100)	None None
Alhissan and Pani [33]	20	3 to 5	Incisors	70; zirconia	24	56/70 (80)	Debonding (18.57%); failure without debonding (1.43%)
Holsinger et al [34]	18	Not reported	Incisors	57; zirconia	20.8*	55/57 (96)	Complete loss of crown (4%)
Bücher et al [35]	667	Not known	Molars or incisors	2388; composite fillings	19	1977/2388 (82.8)	Technical failures (8.3%)
Daou et al [36]	45	6 to 8	Molars	39; PMRC^a^ 37; RMGIC^b^ 35; HVGIC^c^ 38; amalgam	12 12 12 12	39/39 (100) 37/37 (100) 35/35 (100) 38/38 (100)	None None None None

a PMRC: polyacid-modified resin composite.

b RMGIC: resin-modified glass ionomer cement.

c HVGIC: high-viscosity glass ionomer cement.

**Figure 2.**
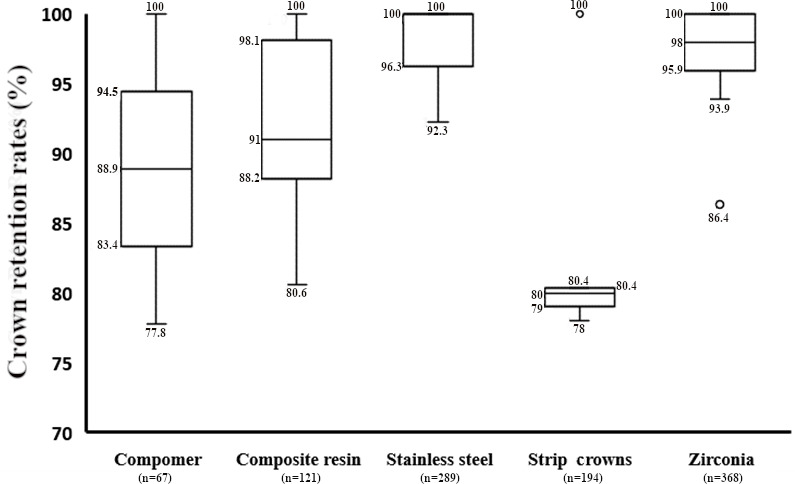
Retention rates for crowns fabricated with different materials, as reported in the included randomized controlled trials.

Figure 2 provides a descriptive analysis of retention rates for crowns fabricated with different materials, as reported in the included RCTs [19-31]. Retention rates varied from 77.8% to 100%, from 80.6% to 100%, and from 92.3% to 100% for compomer, composite resin, and stainless steel crowns, respectively. Additionally, retention rates for strip crowns and zirconia crowns ranged from 78% to 100% and from 86.4% to 100%, respectively. Table 5 illustrates various retentive complications reported in the RCTs for different materials. Regarding clinical studies, debonding and loss of crown were reported for zirconia crowns, while crown fractures were reported for composite resin crowns (Table 4).

**Table 5.** Retentive complications reported in the randomized controlled trials for different materials.

Crown material	Decementation, n	Chipping^a^, n	Crown fracture^b^, n	Poor anatomic form, n	Complete crown loss, n	Partial material loss^c^, n
Compomer	0	0	8	0	0	0
Composite resin	0	12	3	3	1	2
Stainless steel	0	0	0	0	0	0
Strip crowns	0	3	0	0	7	19
Zirconia	5	0	0	0	1	1

a Chipping: loss of small fragments or pieces from the surface of the crown.

b Crown fracture: severe damage where the crown splits or breaks into two or more parts.

c Partial material loss: a more substantial loss of crown material compared to chipping but does not constitute a complete fracture.

### Biological Complications Reported in the Included Studies

Table 6 depicts the distribution of biological complications reported in the included RCTs [18-30]. In the prospective and retrospective clinical studies, 8.3% (6/67) of children receiving compomer crowns and 8.8% (11/121) receiving composite resin crowns reported secondary caries [31-35]. Additionally, 3.3% (10/289) and 21.8% (80/368) of children with stainless steel crowns and zirconia crowns, respectively, in these studies exhibited gingival inflammation [32-36].

**Table 6.** Biological and clinical complications reported in the randomized controlled trials for different materials.

Biological and clinical complications	Compomer (n=67)	Composite resin (n=121)	Stainless steel (n=289)	Strip crowns (n=194)	Zirconia (n=368)
Secondary caries, n (%)	4 (5.97)	3 (2.47)	13 (4.54)	12 (6.18)	0 (0)
Gingival inflammation, n (%)	0 (0)	29 (24.24)	23 (7.85)	14 (7.21)	13 (3.5)
Plaque index^a^, n (%)	0 (0)	0 (0)	0 (0)	0 (0)	25 (6.76)
Bleeding on probing, n (%)	0 (0)	0 (0)	0 (0)	0 (0)	43 (11.77)

a Plaque Index: a clinical measure used to assess the amount of dental plaque on teeth.

### Technical Complications Reported in the Included Studies

Table 7 outlines the distribution of technical complications reported in the included RCTs [18-30]. In the prospective and retrospective clinical studies, 29% (19/67) of compomer crowns exhibited marginal adaptation complications, while stainless steel crowns did not demonstrate marginal adaptation issues [32-36]. No marginal discoloration or plaque retention complications were observed for compomer crowns. Among the 399 zirconia crowns evaluated, 4.8% (19/399) demonstrated shade mismatches, and 1% (4/399) exhibited marginal integrity complications [32-36].

**Table 7.** Percentage of technical complications reported in the included randomized controlled trials [19-31].

Crown material	Surface roughness	Occlusal wear	Marginal adaptation	Marginal discoloration	Marginal integrity	Staining	Plaque retention	Shade mismatch	Opposing tooth wear
Compomer (n=67), n (%)	0 (0)	0 (0)	19 (29)	0 (0)	0 (0)	0 (0)	0 (0)	0 (0)	0 (0)
Composite resin^a^ (n=154), n (%)	2 (1.3)	17 (11)	8 (5.2)	2 (1.3)	0 (0)	24 (15.6)	20 (13)	45 (29.2)	0 (0)
Stainless steel (n=242), n (%)	0 (0)	0 (0)	0 (0)	0 (0)	1 (0.4)	7 (2.9)	0 (0)	0 (0)	33 (13.6)
Strip crowns (n=194), n (%)	0 (0)	0 (0)	14 (7.2)	0 (0)	7 (3.6)	6 (3.1)	0 (0)	41 (21.11)	31 (16)
Zirconia (n=399), n (%)	1 (0.3)	0 (0)	2 (0.5)	0 (0)	4 (1)	7 (1.8)	2 (0.5)	19 (4.8)	34 (8.5)

a Technical complications were reported more for composite resin in the included studies.

### Quantitative Analysis (Meta-Analysis) of the Included Studies

Table 8 presents the estimated 3-year and 5-year retention rates of crowns fabricated using various materials, as reported in the included RCTs [19-31].

The Pearson goodness-of-fit test was performed to assess heterogeneity across studies and to determine whether a fixed-effects or random-effects model should be used. Since no statistically significant heterogeneity was detected in any of the individual groups and rates (*P*>.05), a fixed-effects Poisson regression model was used to estimate the parameters.

**Figure 3.**
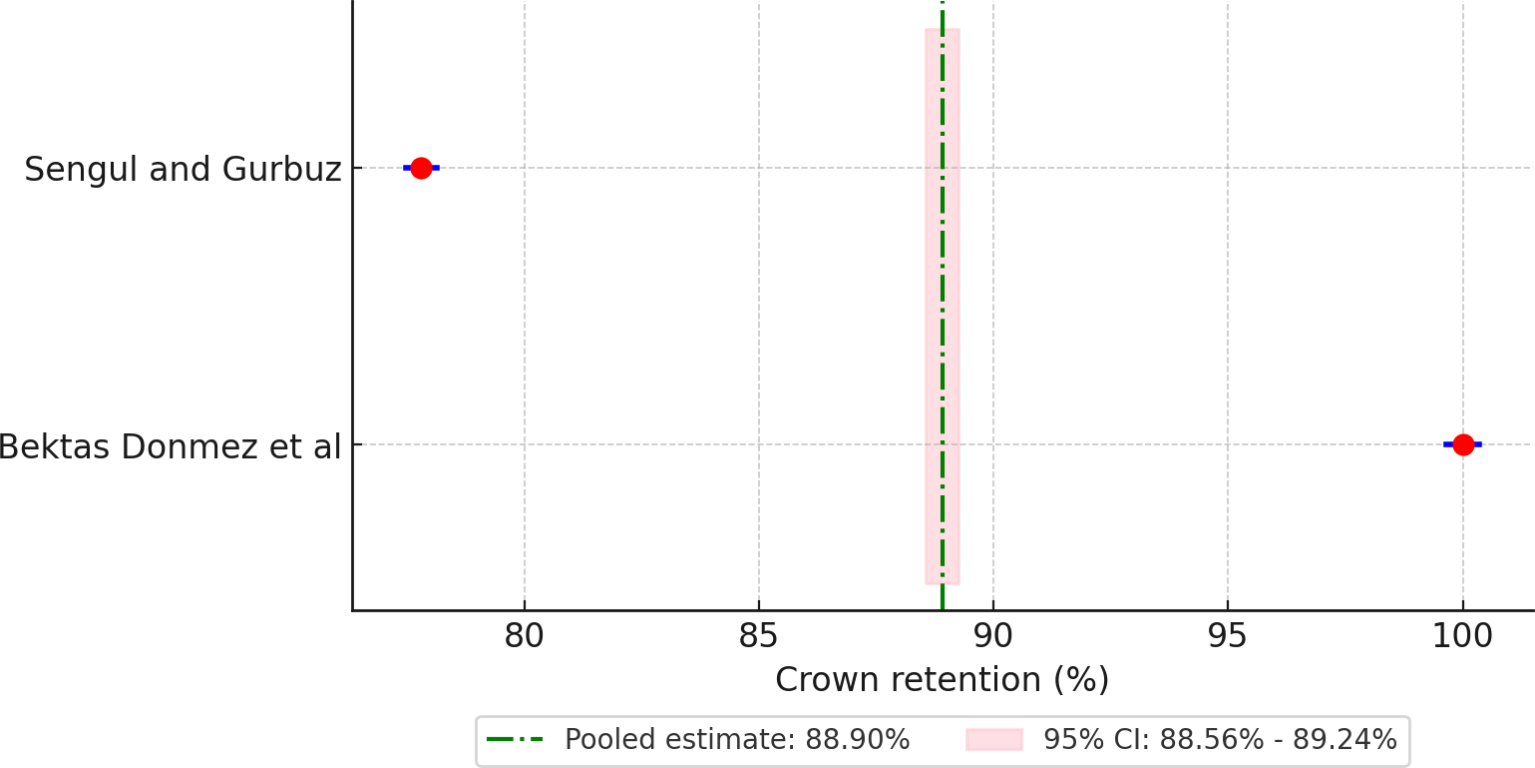
Forest plot illustrating the retention rates of compomer crowns [29,30].

**Figure 4.**
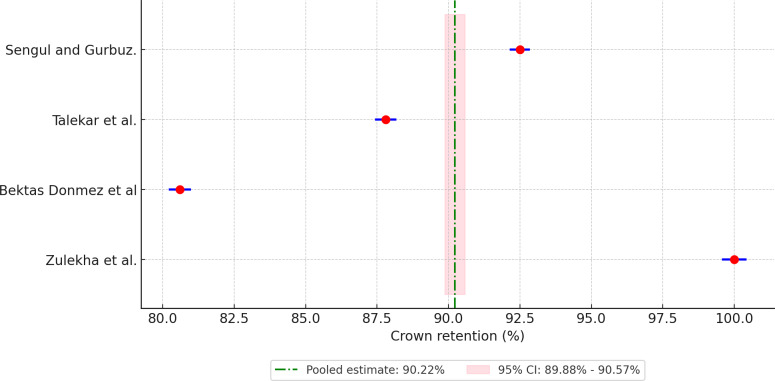
Forest plot illustrating the retention rates of composite resin crowns [19,21,29,30].

**Figure 5.**
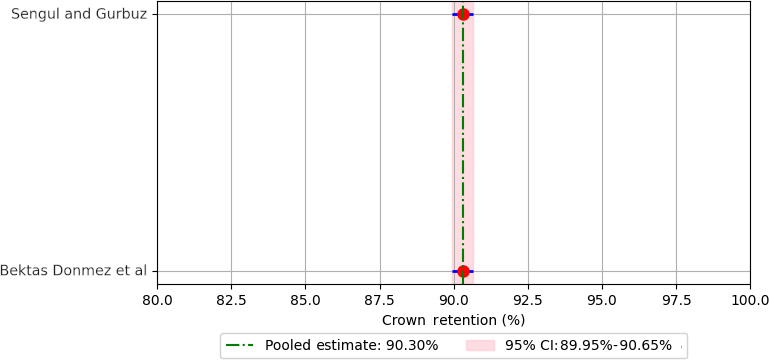
Forest plot illustrating the retention rates of resin-modified glass ionomer cement (RMGIC) crowns [29,30].

**Figure 6.**
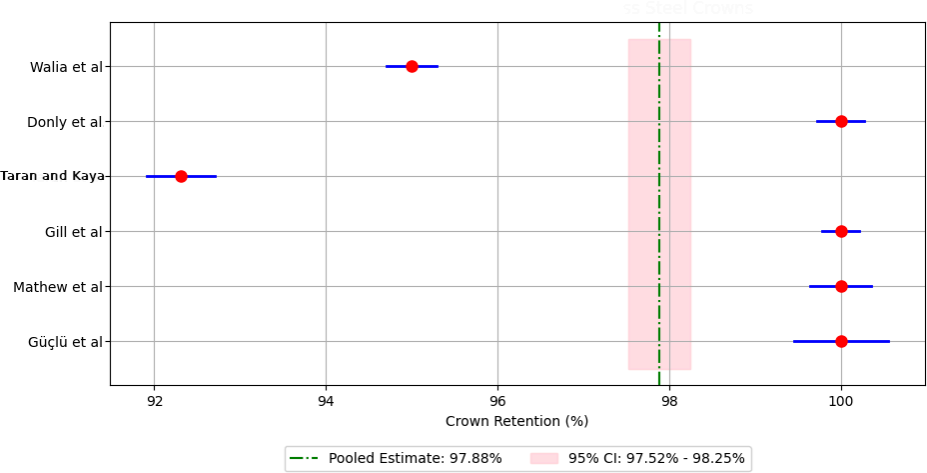
Forest plot illustrating the retention rates of stainless steel crowns [23,25-28,31].

**Figure 7.**
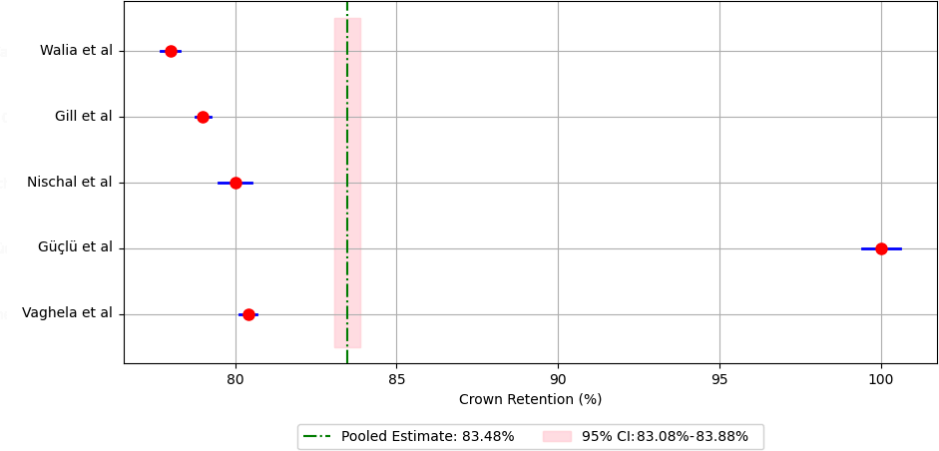
Forest plot illustrating the retention rates for strip crowns [20,23,24,26,31].

**Figure 8.**
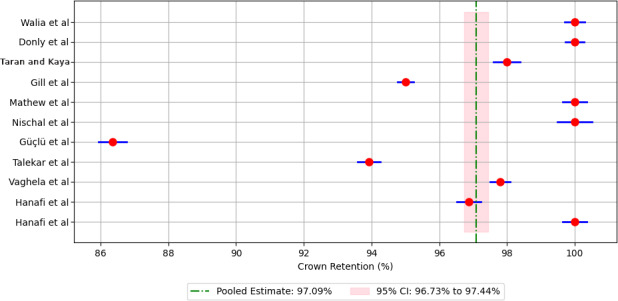
Forest plot illustrating the retention rates for zirconia crowns [20-28,31].

**Figure 9.**
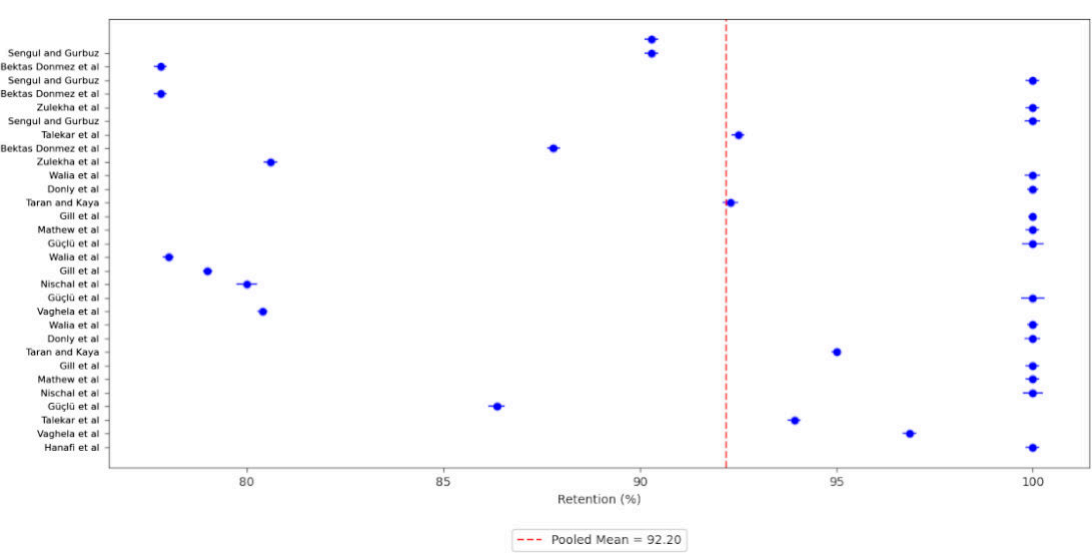
Forest plot illustrating pooled 5-year retention rates across all studies without stratification by crown material [19-31].

Meta-analysis of pooled 5-year retention rates for different crown materials revealed the following retention rates: 88.90% for compomer crowns (Figure 3), 92.18% for composite resin crowns (Figure 4), 90.30% for RMGIC crowns (Figure 5), and 97.88% for stainless steel crowns (Figure 6). Additionally, strip crowns exhibited a retention rate of 83.48% (Figure 7), while zirconia crowns had a retention rate of 97.09% (Figure 8). Figure 9 presents the retention rates, irrespective of the material, and found a retention rate of 92.20% (95% CI 92.14%‐92.26%).

**Table 8.** Estimated 3-year and 5-year crown-retention rates in primary tooth from data reported in the included randomized controlled trials [19-31].

Authors and crown material		Exposure time (days), n	Estimated failure rate per crown year	Estimated 3-year retention rate (%)	Estimated 5-year retention rate (%)
Zulekha et al [19]					
One-shade composite resin		300	0	100	100
Composite resin		300	0	100	100
Walia et al [31]					
Strip crowns		258	0.42	28.5	12.3
Zirconia		258	0	100	100
Stainless steel		258	0.09	75.7	62.8
Vaghela et al [20]					
Strip crowns		495	0.24	48.3	29.8
Zirconia		423	0.03	91.8	86.8
Taran and Kaya [27]					
Stainless steel		312	0	100	100
Zirconia		312	0.08	79.3	68.1
Talekar et al [21]					
Glass-reinforced resin		594	0.28	42.8	24.3
Zirconia		594	0	100	100
Sengul and Gurbuz [30]					
Hybrid composite resin		960	0.04	89.4	82.9
RMGIC^a^		768	0.05	86.9	79.1
Compomer		864	0.11	71.7	57.4
Giomer		912	0.05	85.3	76.9
Nischal et al [24]					
Strip crowns		135	0.18	58.7	41.1
Zirconia		135	0	100	100
Luxa		135	0.18	58.7	41.1
Mathew et al [25]					
Zirconia		1080	0	100	100
Stainless steel		1080	0	100	100
Hanafi et al [22]					
Zirconia		186	0	100	100
Zirconia		192	0.06	82.9	73.2
Güçlü et al [23]					
Strip crowns		66	0	100	100
Zirconia		132	0.27	44.1	25.6
Stainless steel		78	0	100	100
Gill et al [26]					
Strip crowns		840	0.13	68	52.6
Zirconia		840	0.01	95.8	93.1
Stainless steel		960	0	100	100
Bektas Donmez et al [29]					
RMGIC		558	0.06	82.4	72.4
Compomer		558	0	100	100
Composite resin		558	0.06	82.4	72.4
Donly et al [28]					
Zirconia		1200	0	100	100
Stainless steel		1200	0	100	100

a RMGIC: resin-modified glass ionomer cement.

## Discussion

This systematic review examined data from RCTs and prospective and retrospective clinical studies to evaluate the performance of dental crowns for primary teeth. Key findings include significant biases in RCTs affecting the assessment of crown efficacy, variability in age distribution impacting retention rates, and varying complications across different crown materials. Despite these challenges, stainless steel crowns consistently demonstrated superior retention rates, while complications such as secondary caries and gingival inflammation varied among materials.

**Figure 10.**
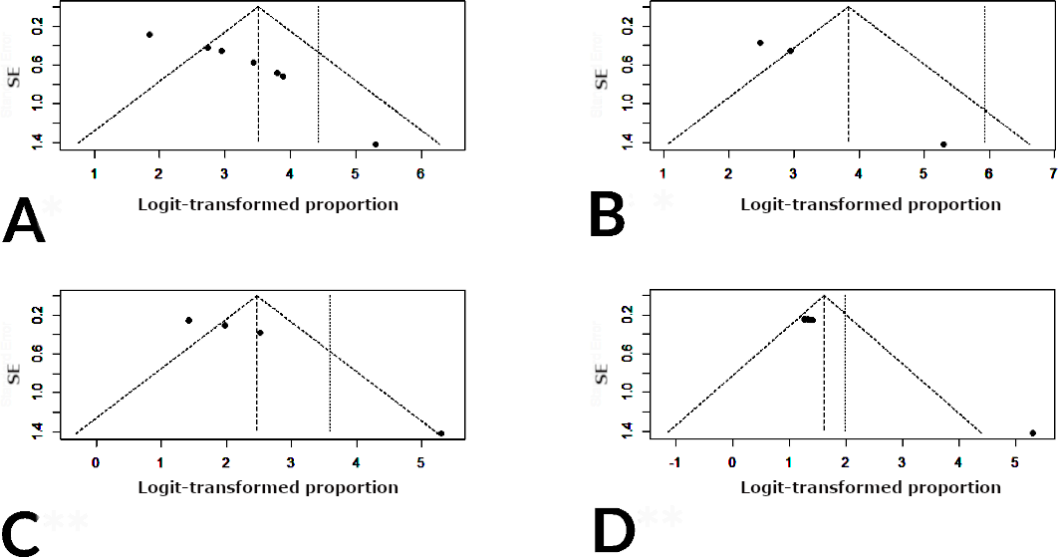
Funnel plots for assessments of publication bias in the included studies for (A) zirconia, (B) stainless steel, (C) composite resin, and (D) strip crowns. For part A, the Egger test was performed (*t*_9_=9.07, *P*<.001). For parts B-D, the number of studies was below the recommended threshold for robustly assessing publication bias, and the potential for small study effects should be considered with prudence.

In our analysis, stainless steel crowns exhibited the highest retention rates, corroborating findings from previous studies [8,10], which also highlighted the superior performance of stainless steel crowns in terms of retention and durability. The study by Chisini et al [8] indicated an annual failure rate between 0% and 29.9%, with stainless steel crowns showing the highest success rate of 96.1%. In this study, the estimated 3-year and 5-year retention rates ranged from 75.7% to 100% and from 62.8% to 100%, respectively. Zirconia and composite resin crowns also demonstrated high retention rates, although they were associated with different types of complications. These results align with another study by Alzanbaqi et al [9], which noted improved gingival and periodontal health, excellent retention, and high fracture resistance with zirconia crowns for primary teeth. Regarding complications, strip and composite resin crowns showed higher instances of chipping and partial material loss, likely due to the inherent brittleness of these materials and their susceptibility to fracture under masticatory forces [37-39]. Crowns fabricated with composite resins and newer materials like compomer exhibited a higher incidence of fractures, possibly due to inadequate bonding strength and less favorable mechanical properties compared with stainless steel and zirconia [39,40]. Composite resin crowns also exhibited higher rates of gingival inflammation, likely due to plaque accumulation and potential marginal leakage. Conversely, zirconia crowns showed minimal gingival inflammation, attributed to their smooth surface and biocompatibility [40-42]. Secondary caries was a significant issue across all materials but was particularly prevalent in crowns with poor marginal adaptation, emphasizing the need for precise placement and proper maintenance to mitigate caries risk [42,43]. While the findings demonstrate the efficacy of stainless steel crowns, material choice should consider the specific needs and conditions of pediatric patients. Clinicians must weigh the benefits of high retention rates against the potential for biological complications. The included RCTs displayed a notable prevalence of allocation concealment, performance bias, and detection bias (Table 1) [19-31]. These biases, inherent to the nature of interventions that often preclude optimal double blinding, necessitate careful consideration in assessing the risk of bias for studies involving crowns for primary teeth. These findings align with a previous systematic review by Chisini et al [8], which also identified a high risk of bias. The assessment of publication bias in our meta-analysis revealed noteworthy findings (Figure 10). The Egger test for zirconia crowns suggested potential publication bias. In contrast, for stainless steel, composite resin, and strip crowns, the limited number of studies precluded robust evaluation of small study effects. Thus, caution is warranted in interpreting these outcomes due to the small sample size. The significant results indicate asymmetry in the funnel plot, potentially influencing effect size estimates.

The age distribution of the study population is often not reported, posing challenges in determining optimal retention rates for a smooth transition from primary to permanent teeth, especially in younger age groups. The age of children in the RCTs and prospective and retrospective clinical studies ranged from 1 to 10 years and from 3 to 10 years, respectively [19-31]. The reported crown-retention rates for different age groups suggest potential nuances in the transition from primary to permanent teeth, underscoring the need for future studies to provide detailed age-distribution data. Another major limitation of this review is the varying sample sizes across studies, which could influence the generalizability of the results. However, the application of Poisson regression allowed for the adjustment of these differences, providing a more accurate comparison of retention rates [44]. Despite these limitations, this review provides a comprehensive analysis of crown retention and associated complications in primary teeth, offering valuable insights for evidence-based clinical decision-making.

Future studies should aim to include larger sample sizes and longer follow-up periods to validate the findings of this review. Additionally, research should focus on the development and evaluation of new materials and techniques that could enhance crown retention and reduce complications. Investigating advanced fabrication methods such as 3D printing and computer-aided design and computer-aided manufacturing technology holds promise for improving the fit and longevity of dental crowns.

In conclusion, while stainless steel crowns remain a gold standard for primary teeth restorations due to their high retention rates and durability, the choice of material should be tailored to individual patient needs, considering both mechanical properties and potential biological complications. Ongoing research and clinical advancements will continue to refine these treatment modalities, ultimately improving outcomes for pediatric dental patients.

## Supplementary material

10.2196/57958 Multimedia Appendix 1 Search syntax used for searching databases.

10.2196/57958 Checklist 1 PRISMA (Preferred Reporting Items for Systematic Reviews and Meta-Analyses) checklist 2020.

## Abbreviations

MOOSE: Meta-Analysis of Observational Studies in Epidemiology
PICOS: Population, Intervention, Comparison, Outcomes, and Study design
PRISMA: Preferred Reporting Items for Systematic Reviews and Meta-Analyses
PRISMA-P: Preferred Reporting Items for Systematic Review and Meta-Analysis Protocols
RCT: randomized controlled trial
RMGIC: resin-modified glass ionomer cement
