# Supplementary material for: Reasons for Crown Failures in Primary Teeth: Systematic Review and Meta-Analysis
Source: Interact J Med Res. 2025 May 1;14:e57958. doi: 10.2196/57958 (PMC12061352; doi:10.2196/57958)
Supplement: Multimedia Appendix 1 [file ijmr-v14-e57958-s001.docx]

**Appendix 1: Search Syntax Used for Searching Databases**

("Pediatric dentistry" OR "Pediatric dentists" OR "Pedodontics" OR "Children's dentistry") AND ("Primary teeth" OR "Deciduous teeth" OR "Baby teeth" OR "Primary dentition") AND ("Crowns" OR "Dental crowns" OR "Tooth crowns" OR "Restorations") AND ("Failure" OR "Complications" OR "Reasons" OR "Causes" OR "Outcomes") AND ("Randomized controlled trial" OR "RCT" OR "Observational study" OR "Cohort study" OR "Case-control study")
